# Listening to patients: A qualitative study on diagnostic delay, coping strategies and stigma in early‐onset colorectal cancer

**DOI:** 10.1111/codi.70285

**Published:** 2025-10-28

**Authors:** H. L. Gros, S. Taha‐Mehlitz, S. Erdem, M. Worni, M. Henschel, A.‐K. Huber, J.‐M. Gass, A. Müller, C. T. Viehl, R. Galli, D. Rodjakovic, T. Simon, S. Happ, S. Teixeira da Cunha, L. Eisner, D. Steinemann, B. Müller‐Stich, M. von Strauss und Torney, J. M. Klasen

**Affiliations:** ^1^ Department of Surgery Clarunis University Digestive Health Care Centre Basel Switzerland; ^2^ Klinik für Viszeralchirurgie Bern Switzerland; ^3^ Hirslanden Klinik Beau‐Site Bern Switzerland; ^4^ Klinik für Allgemein‐ und Viszeralchirurgie, Luzerner Kantonsspital Luzern Switzerland; ^5^ Department of Health Sciences and Medicine Universität Luzern Luzern Switzerland; ^6^ Chirurgische Klinik, Spitalzentrum Biel Biel Switzerland; ^7^ Klinik Chirurgie & Viszeralchirurgie, Kantonsspital Baselland Liestal Switzerland; ^8^ Klinik für Chirurgie, Spitalzentrum Oberwallis Visp Switzerland; ^9^ Departement Chirurgie Kantonsspital Olten Olten Switzerland; ^10^ University of Basel Basel Switzerland; ^11^ St. Clara Research Ltd Basel Switzerland

**Keywords:** coping strategies, diagnostic delay, early‐onset colorectal cancer, patients perspective, qualitative research, stigma

## Abstract

**Aim:**

Early‐onset colorectal cancer (EOCC), defined as colorectal cancer (CRC) diagnosed in patients under the age of 50, has increased alarmingly in Western countries in recent decades. Although risk factors, diagnostics and treatment approaches have been studied, the subjective experiences of EOCC patients remain underexplored. This study aimed to identify patient‐perceived challenges and their unmet needs in Switzerland, a country with a high‐resource healthcare system with minimal waiting times for investigations and comprehensive healthcare coverage.

**Method:**

Nineteen semi‐structured interviews were conducted between 09/2023 and 06/2024 with EOCC patients following a constructivist grounded theory approach.

**Results:**

Participants from all cancer stages and aged between 30 and 50 years were recruited. We developed three main themes from the collected data: diagnostic delay, coping mechanisms and stigmatisation. Despite access to a well‐resourced healthcare system, study participants experienced diagnostic delays, indicating the need for increased physician awareness and improved risk‐based screening for high‐risk individuals. Participants' key coping strategies included self‐advocacy and participating in medical decision‐making, like prioritising fertility preservation over rushed treatment. Participants reported fear of stigmatisation and isolation after being diagnosed. Such stigma manifested in concerns over alopecia or living with an ostomy. Finally, participants experienced an internal shift in personal priorities resulting in post‐traumatic growth despite cancer diagnosis

**Conclusions:**

EOCC challenges current clinical practices. Better physician awareness, risk‐factor‐based screening policies, integrated discussions about individual treatment such as potential fertility issues and targeting stigma are critical for improving the EOCC patients' journey.

**What does this paper add to the literature?**

This paper adds evidence on EOCC patients' experiences within a high‐income country with universal health insurance coverage. It identifies relevant provider misdiagnosis as a contributor to diagnostic delays, reveals coping strategies and post‐traumatic growth—offering findings transferable to countries that share comparable healthcare structures and socio‐economic conditions.

## INTRODUCTION

Early‐onset colorectal cancer (EOCC), defined as colorectal cancer (CRC) diagnosed before age 50, has risen alarmingly in most Western countries over recent decades [1, 2, 3, 4]. Recent studies have associated EOCC with lifestyle factors prevalent in Western societies, such as obesity, sedentary behaviour, high‐fat diets and consumption of sugar‐sweetened beverages [5]. Prolonged antibiotic exposure may disrupt the gut microbiome and contribute to CRC development [6]. Additionally, a recent international study suggests that exposure to the bacteria‐produced mutagen colibactin could contribute to the global increase of EOCC [7]. While inherited syndromes are present in a minority of EOCC cases, most are sporadic and not explained by genetic factors alone [6]. By 2030, colon and rectal cancer incidence rates are projected to rise by 90% and 124%, respectively, in adults aged 20–34 and by 28% and 46% in those aged 35–49 [8]. Moreover, EOCC patients are diagnosed in higher stages, leading to more aggressive treatments and potentially fatal outcomes [9]. These data emphasise the need to adapt public health recommendations to disease trends [10, 11]. In 2020, the ‘Never Too Young Survey Report’ (884 EOCC patients, median age 42) found that 54% were initially misdiagnosed, 36% consulted three or more doctors before diagnosis and full‐time employment fell from 75% at diagnosis to 50% by the survey time [12]. This highlights two main issues: a healthcare system knowledge gap on EOCC and a significant socio‐economic impact. To identify further needed considerations from the lowered diagnosis age, some qualitative studies explored EOCC patients' cancer journey. Unmet needs were found in financial burden, quality of life and fertility [13, 14, 15, 16, 17]. Despite these, research remains focused on objective outcomes like risk factors, diagnostics and therapies [18, 19], while gaps persist in understanding EOCC patients' perceptions, challenges and unmet needs [20]. Additionally, to our knowledge, no qualitative study on the cancer journey and lived experience of EOCC patients has been performed in a high‐income country with a high‐resource healthcare system, minimal waiting times for investigations and comprehensive healthcare coverage [21]. Therefore, it seems an intriguing opportunity to explore how diagnostic delays, psychosocial burdens and patient experiences unfold even in optimal systemic conditions—highlighting barriers that may be independent of healthcare infrastructure and rooted in clinical awareness, societal perceptions or individual trajectories. This qualitative study—as part of a larger quantitative multicentre study in Switzerland on EOCC patients—aimed to bridge these gaps by shifting the focus from clinical outcomes to EOCC patients' experiences and perceptions [22]. After identifying areas that require improvements, we propose a series of actions.

## METHODS

### Study design

This research followed the constructivist grounded theory approach to explore participants' in‐depth experiences of their CRC journey, and how they interpreted and understood their perceptions [23]. Data were gathered via semi‐structured interviews, followed by iterative qualitative coding and constant comparison to identify recurring themes. Memo‐writing and field notes supported dynamic interaction between the research team while discussing the data [23]. The research design used the Standards for Reporting Qualitative Research checklist for comprehensive reporting [24].

### Recruitment and participants

This qualitative study was conducted as part of a larger multicentre study across 11 Swiss hospitals, where 764 participants were prospectively enrolled between 01 January 2023 and 30 June 2024 [22]. This quantitative study explored differences in clinical profiles, socio‐economic factors and oncological treatments between EOCC and later‐onset colorectal cancer patients [22].

We used expert purposive sampling with a target sample size of 15–20 participants: Surgeons at each site were informed about the qualitative study part and invited to recruit information‐rich participants most likely to provide deep insights to answer the research questions. This multisite recruitment strategy ensured that regional and institutional differences were represented [25]. After obtaining consent, JG scheduled the interview. Participants chose a setting comfortable for their needs (in‐person or virtual meetings), with research team support available anytime. Participants came from various regions and hospitals of different sizes, while all cancer stages were included (Table S1). Eligible participants were diagnosed with CRC between ages 18 and 49, provided informed consent, and were fluent in German or French to be able to follow a 60‐minute semi‐structured interview.

### Data collection and analysis

The research team (MVS/ST/SE/HG/JK) developed the semi‐structured interview guide (Supporting Information). Questions were developed based on reported unmet needs and through prior clinical experiences with EOCC patients. Most questions were open‐ended to facilitate in‐depth responses [23]. Interviews were conducted by JG or HG (for French‐speaking patients) between 21/09/2023 and 26/06/2024. Interviews were audio‐recorded and transcribed verbatim, supported by AI software. We reached data sufficiency after 17 interviews and two additional interviews confirmed data saturation [23, 26, 27]. Anonymity was ensured using non‐identifiable codes. The research team used Quirkos for data management and analysis [28]. HG and ST, trained by JK, first jointly coded one interview to harmonise coding practice, then independently coded four transcripts each. HG/ST subsequently coded one interview jointly, reviewed previous coding and independently coded a further four (ST) and five (HG) transcripts. In regular meetings, codes and developed themes were discussed (HG, ST and JK) to reduce inter‐coder and individual bias, clarify ambiguities and strengthen interpretive rigour. Memo‐writing, used as a ‘thinking space’ of this study (especially used by HG and ST)—documented the researchers' team analytic journey, enhanced the reflexivity process and provided the scaffolding for credible, rigorous interpretation. To ensure methodological rigour, we followed established criteria of credibility, dependability, confirmability and transferability [29]. The use of dual coding, iterative discussions and reflexive field notes, as well as memo‐writing strengthened the credibility and trustworthiness of the findings. We documented analytic decisions to support dependability and engaged several researchers in dialogue to limit individual bias and enhance confirmability. Transferability was facilitated through rich description of participant contexts and illustrative quotations. Together, these strategies supported keeping the analytic process transparent and grounded in participants' accounts.

## RESULTS

### Participant characteristics

Nineteen participants agreed to an interview of 40 min on average (25–65 min). Participants (aged 30–50) were recruited from seven institutions (Table S1).

### Topics

We identified three main themes: diagnostic delay, coping mechanisms and stigmatisation. Below, we present these findings, highlighted with participants' quotes, while minor themes are available in Figures S1–S7.

#### Diagnostic delay

Participants described symptoms like bleeding, constipation, burning sensation, abdominal cramps or weight loss. Most participants, however, were torn between suspicious perception, ‘knowing something was wrong (P17)’, and trivialisation, searching for other, simpler rationales: ‘… in my head it was always explained somehow. Yes, food and […] stress […] always some excuses […] Looking back now, that wasn't right on my part at all (P11)’. Similarly, another participant explained:

I blamed it on my diet […] and tried to experiment a bit, but it didn't stop…. I was still […] tired […], but I had the feeling that it was […] due to work […] I have a […] demanding work, lots of deadlines, […], a large team that I look after, so I put it down to that. (P13)

Most participants attributed their symptoms to stress or food intolerance. However, as suspicions grew, participants tried to seek support from healthcare providers or relatives. Given their young age, symptoms were frequently mistaken for something benign, leading to a lack of further diagnostic evaluation, even when physicians were explicitly asked about colonoscopy, highlighted by this example: ‘I told the family doctor I want to do a colonoscopy. And she said, “no”, that's unnecessary, you're not 50 yet, it's only free from then on (P09)’. Similar voices from the private environment also mitigated potential symptoms: ‘Many people said, ‘no, don't do a colonoscopy’, you can still do it when you're 50. I sometimes had that [kind of symptom] for half a year, it passes (P18)’ or ‘… my boyfriend said that he'd had blood come out of his bottom before, a haemorrhoid, and that it would go away (P20)’.

Participants were often first treated for benign proctological diseases if bleeding was present (P07/P19/P12/P02/P06/P20—Figure S1). Abnormal results like ‘quite deep iron level (P04/P20)’, ‘increased inflammatory markers in the blood (P08)’ or ‘a slightly positive stool sample (P09)’ did not lead to immediate further diagnostics either. If no bleeding occurred but bowel movement changes were present, stress reduction was suggested: ‘I saw three different doctors, and they all thought it was due to stress because I had a lot of mental pressure at work (P14)’. Overall, participants felt their symptoms were dismissed despite their concerns and active efforts to seek help from family doctors. Reluctance to pursue further diagnostics beyond common differential diagnoses resulted in loss of confidence, as one participant highlighted:

When I went the second time with the symptoms, without pain, just blood on the stool […] I also told him that this is extreme and then still not being taken seriously, I don't understand that. I changed to another doctor for this reason. (P06)

Therefore, participants (P03/P20/P16) agreed that ‘doctors should be better trained, so you don't have to visit ten times before getting a diagnosis (P16)’.

For participants with less proactive healthcare providers, diagnostic steps were initiated after several months of symptom misinterpretation, often due to pressure coming from the patients themselves: ‘… I went back to him two weeks later and told him: please refer me now [for a colonoscopy] (P06)’. Another confirmed this reaction: ‘I said that something was wrong. I would be happy if they could do another examination (P12)’. Participant 16 had to be even more persistent to learn about their diagnosis:

I went to the doctor three times. And then I said, […] I'm not going out until I know what I have. [The doctors] checked and did everything […] then, they saw black dots on the liver during ultrasound. (P16)

Further diagnostic delay followed due to colonoscopy scheduling difficulties: ‘Unfortunately, the appointment for the colonoscopy wasn't until five weeks later (P07)’.

In conclusion, all participants agreed on colonoscopy being the most reliable method to confirm CRC diagnosis. This conviction was so strong that one participant stated, ‘I would rather have a colonoscopy once a week (P03)’.

#### Coping through support‐seeking and regaining control

While CRC diagnosis shocked most participants, their families and healthcare providers, it also brought relief by finally providing an answer and restoring control. One participant was desperate for a diagnosis, even a bad one: ‘I was able to calm [the doctor] down and told her I came here with the wish something would be found. So, it would stop, something would change (P13)’.

Openly sharing diagnosis helped participants cope: ‘I told [all my family] as soon as I got home […] I told everyone, my colleagues (P09)’ or ‘I told a lot of people, including closest friends, work colleagues, the entire team and supervisor (P05)’. By verbalising their experience, participants sought emotional support and regained control.

Encouraging family members to undergo colonoscopies was also identified as coping, supporting the concept that when individuals feel unable to help themselves, they seek to help others. In doing so, they channelled their anguish into action, finding meaning in facilitating preventive care for loved ones. For instance, one participant ‘… set up a little WhatsApp group [with friends]. Some of them didn't have cancer, but the first preliminary stages were found. And they were all under 50. So […] out of 20, maybe 12 had something that needed to be treated (P03)’.

How CRC diagnosis was communicated significantly influenced participants' coping. Since diagnosis typically follows a colonoscopy, patients were usually informed in person, often with a relative present (Figure S2), who provided immediate emotional support. However, receiving a cancer diagnosis alone, ‘I could no longer understand the world. My world collapsed. (P16)’, over the phone: ‘I don't think that's human. There are things you don't do on the phone […] I just wanted to get away from there. (P07)’ or being presented with a bleak future ‘my family doctor [said] it was bad. I felt like I was going to die tomorrow (P16)’ led to loss of doctor‐patient trust, prompting a change of physician to restore control over their care.

After diagnosis, some participants struggled with the frustration of ‘Waiting. Do nothing and wait (P04)’, adopting proactive behaviours driven by anxiety of tumour progression and fear of being neglected by their healthcare provider, especially after experiencing diagnostic delay (Figure S3). Conversely, others struggled with the rapid succession of medical appointments (Figure S4). Participants also recalled difficulties advocating for fertility preservation (Figure S5), with some prioritising family planning over rushed cancer treatment.

#### Living with cancer: From stigma to post‐traumatic growth

Some patients sought to explain their diagnosis by drawing on general knowledge around lifestyle factors like meat consumption, smoking and/or obesity, as one participant illustrated:

You see people sitting there, incredibly overweight. A cigarette in their hand […] curry sausage on the table. And then you think, hey, it should have been you and not me. […], 'Man, it should have been your turn and not me… You see them […], pulling the black burnt meat off the grill. (P03)

Cancer patients perceived societal judgement and feared being identified as ‘sick’ by others: “Go shopping without a shower, […], in the middle of the day, you could imagine I wasn't working, I was sick. (P05)”. Stigmatisation may have been reinforced as participants felt isolated, having no opportunity to ‘actually discuss with anyone who also had the diagnosis (P20)’.

Fear of stigma manifested in concerns over alopecia and ostomy. One‐third of participants (both male and female: P08/P01/P07/P04/P02/P14/P17—Figure S6) expressed relief as they ‘didn't lose any hair (P07)’. Compared to alopecia, ostomy was easier to hide, leading to less stigma and fewer restrictions. For instance, one participant ‘was able to hide the stoma bag well under my clothes. Depending on what you were wearing […] If you emptied it regularly, you didn't even see it. […] I didn't restrict myself much […], I went to the pool […] (P01)’.

Living with cancer triggered an internal shift, prompting participants to reassess priorities. Participants' lives were disrupted, making it difficult to balance careers while caring for children and elderly family members. Many shielded their children to protect them emotionally and maintain normalcy (Figure S7).

Many eventually prioritised ‘making time for myself (P11)’. One participant reflected, ‘I think I need to concentrate on myself […]. I want to get something out of life. And not just work and be a mom and not be me anymore (P04)’. Most participants engaged in outdoor activities for their well‐being or emphasised daily activity, saying: ‘I made sure that I still got plenty of exercise. I went for long walks […] during therapy (P06)’. Others turned to spirituality and alternative medicine: ‘I do shamanic journeying, meditation and singing bowls (P10)’.

While participants used above‐mentioned strategies to adapt, returning to employment was key to regaining ‘a kind of everyday life (P17)’ and allowing social interactions, helping them to feel connected to their pre‐diagnosis life: ‘No, I don't have any new goals. I just want to get back to work 100% (P06)’. Another participant described the resulting emotional toll of full sick leave:

I'm no longer working […] That's hell for me. Just being at home […] I have to make sure that I don't fall into a hole and then start […] brooding again. Because work and being busy is distracting. (P04)

In conclusion, while external stigma reinforced isolation, it also fostered self‐prioritisation. Many tried to stay busy to have the feeling of control. Returning to work was crucial, providing both distraction and a path to normalcy.

## DISCUSSION AND CONCLUSIONS

This qualitative exploration of EOCC patients' challenges and unmet needs provides insights for improving patient‐centred care [13, 14, 15]. It adds new evidence on the cancer journey and lived experiences of EOCC patients in a high‐income country with universal health coverage and minimal waiting times for investigations [21]. While set in Switzerland, our findings may be transferable to neighbouring European countries and other high‐income countries with similar healthcare and socio‐economic systems [30, 31, 32].

Our study confirmed that provider misdiagnosis contributes to diagnostic delays in EOCC patients despite the strengths of the Swiss healthcare system [12, 14, 16, 21]. Participants suggested lowering the screening age and improving physician awareness to reduce delays. While the US and Japan now start CRC screening at 45 and 40, respectively, we must analyse whether this approach would suit countries where screening starts at 50 [10, 33, 34, 35]. In Switzerland, only 15 of 26 cantons offer organised screening for individuals over 50 and lowering the age would increase colonoscopy demand by 12%–23%, raising cost‐utility concerns [36, 37, 38]. Therefore, raising physician awareness and improving risk‐based screening for high‐risk individuals may be preferable to lowering the screening age [34, 36]. Participants' frequent attributions of symptoms to stress and food intolerance illustrate how, in the absence of clear, standardised frameworks for healthcare providers (such as the UK's ‘Getting It Right First Time’, which includes a dedicated CRC diagnostic pathway aiming to confirm or exclude a diagnosis within 28 days of referral [39]), patients attempted to fill the knowledge gaps themselves, relying on personal reasoning or advice from peers and social media. Stanich et al. found that 16.4% of EOCC cases would have been preventable with adherence to high‐risk guidelines, while Gupta et al. showed 98.4% should have been advised screening before their diagnosis age [34, 40, 41]. Incorporating EOCC‐related training into Switzerland's continuing medical education may address this gap [42]. Despite low physician awareness, EOCC is gaining attention in mainstream media, including *Grey's Anatomy* and *Time* [43, 44]. Further strategies to reduce diagnostic errors, suggested by Abimanyi‐Ochom et al., include computerised trigger and/or decision support systems [45]: scanning patient data for indicators (e.g., abnormal tests and repeated visits), alerting clinicians or initiating follow‐up protocols might decrease these delays.

Following diagnostic delay, participants coped by sharing their experiences with family and encouraging relatives to undergo colonoscopies. However, lowering screening thresholds without clear guidelines or solely based on peers' recommendations may increase colonoscopy demand, potentially delaying access for high‐risk individuals [36, 37, 38]. A similar trend is visible on social media, where EOCC patients use platforms like TikTok to raise awareness and promote early detection [46]. Such self‐advocacy supports coping and improves quality of life, as seen in cancer patients aged 18–35 or women with advanced gynaecological malignancies [47, 48]. Self‐advocates adapt to life with cancer by establishing a ‘new normal’, integrating it into their lives rather than seeing it as a temporary disruption [49].

Self‐advocacy was crucial in medical decisions, especially fertility preservation, as some prioritised family planning over rushed cancer treatment. With CRC increasingly affecting younger individuals and first‐time parenthood occurring later, reproductive health discussions are often overlooked [1, 2, 50, 51]. To address this issue, we propose integrating fertility counselling into standard pre‐operative care. Research on other cancer types shows that psycho‐educational therapy and multidisciplinary infertility counselling improve psychological outcomes and reduce long‐term regret [52, 53, 54].

Stigmatisation of CRC arises from stereotypes linking the disease to unhealthy lifestyle or anal sex [1, 34, 55, 56]. This illustrates the ambiguous role of identifying risk factors, which patients may perceive as ‘blame factors’. As patients attempt to fill the knowledge gap regarding the cause of their CRC diagnosis, they often draw on societal narratives. In doing so, they may attribute fault to themselves and experience feelings of stigma. However, when they do not exhibit these risk factors, they are left without an explanation and may experience cognitive dissonance. Notably, most EOCC patients lack established lifestyle‐associated or hereditary risk factors yet develop cancer at a young age. Self‐advocacy helped participants overcome cancer stigma, ultimately resulting in PTG, where survivors find new meaning, develop deeper relationships or inner strength [57]. While studies reported that cancer patients experience mental health or financial hardships [14, 15, 58, 59, 60, 61], our participants described PTG [57]. Current literature attributes this positive change to effective healthcare communication, physical activity, employment and access to psychological care at diagnosis [62, 63].

Unlike literature linking ostomy to reduced quality of life and emotional distress [15, 64], our study found it less concerning. Instead, alopecia emerged as a greater issue for young adults of both sexes, at least in our participants. This echoes findings in gynaecological malignancies, where the psychological burden of alopecia can exceed that of mastectomy [65]. However, this observation is novel in the context of CRC, and studies on alopecia are usually focused on female experiences. Physicians often underestimate the significance of alopecia because it is not life‐threatening, with emphasis instead placed on treatment efficacy [65, 66]. Yet psychological stress from hair loss can reduce patients' motivation to continue treatment. Incorporating alopecia management (for example, offering scalp cooling or financial support for wigs [65]) where hair loss is expected or informing patients in early treatment stages when chemotherapy is unlikely to affect their hair could improve patients' quality of life, reduce cancer‐related stigma, alleviate fears and ultimately increase compliance.

In conclusion, despite a well‐resourced healthcare system, diagnostic delay persisted, highlighting the need for improved awareness. Further research is needed to identify effective strategies to enhance self‐advocacy.

### Strengths and limitations

Prospective selection during treatment, rather than retrospective internet‐based recruitment, enabled real‐time sharing of experiences and minimised selection bias [14, 15]. Surgeon‐mediated recruitment of voluntary participants potentially led to the selection of participants eager to share strong positive (like PTG) or negative experiences. Moreover, this kind of recruitment can introduce gatekeeper bias through the selection of patients who reflect the recruiters' expectations of patients' challenges and perceived barriers. This may have shaped the narratives represented and should be considered when interpreting our findings. To mitigate the risk, we recruited from seven institutions, and the interview guide, developed by the core research team, was not shared with the local recruiters, and the interviews were mainly conducted by an independent study nurse.

The interviews only captured EOCC patients' perspectives, omitting healthcare provider insights. Diagnostic delays may be due to system inefficiencies, cost constraints, limited consultation time or fragmented patient histories [67]. Interviewing healthcare providers could help characterise further where diagnostic delays arise and thereby guide efforts to target these areas.

Because participants could choose virtual or in‐person interviews, we must consider potential implications of the remote format. Virtual interviews may create a sense of distance, increase distractions from the home environment and cause fatigue [68]. Some participants may also be distracted by seeing themselves on screen, and it is harder for interviewers to recognise and respond to emotional distress remotely [68].

Quotes have been translated from German/French, potentially resulting in language inaccuracies [69]. Original quotes are available upon request.

## CONCLUSION

The rise of EOCC in Western countries challenges clinical practice, leading to diagnostic delays, even in well‐resourced healthcare systems. We, therefore, need to improve physician awareness through targeted education. We need risk‐factor‐based screening policies for early detection and prevention.

## AUTHOR CONTRIBUTIONS

**H. L. Gros:** Conceptualization; data curation; formal analysis; methodology; visualization; writing – original draft; resources; investigation. **S. Taha‐Mehlitz:** Conceptualization; data curation; formal analysis; funding acquisition; investigation; methodology; resources; supervision; writing – original draft. **S. Erdem:** Conceptualization; data curation; methodology; resources; writing – review and editing. **M. Worni:** Resources; data curation; writing – review and editing. **M. Henschel:** Resources; writing – review and editing; data curation. **A.‐K. Huber:** Data curation; resources; writing – review and editing. **J.‐M. Gass:** Data curation; resources; writing – review and editing. **A. Müller:** Data curation; resources; writing – review and editing. **C. T. Viehl:** Resources; data curation; writing – review and editing. **R. Galli:** Resources; writing – review and editing; data curation. **D. Rodjakovic:** Data curation; resources; writing – review and editing. **T. Simon:** Writing – review and editing; data curation; resources. **S. Happ:** Data curation; resources; writing – review and editing. **S. Teixeira da Cunha:** Resources; writing – review and editing; data curation. **L. Eisner:** Resources; data curation; writing – review and editing. **D. Steinemann:** Data curation; resources; writing – review and editing. **B. Müller‐Stich:** Data curation; resources; writing – review and editing. **M. von Strauss und Torney:** Data curation; conceptualization; funding acquisition; investigation; methodology; project administration; resources; supervision; validation; writing – review and editing. **J. M. Klasen:** Conceptualization; formal analysis; investigation; methodology; resources; supervision; validation; writing – review and editing.

## FUNDING INFORMATION

This study was funded by the Basel Cancer League (KLbB 5839‐02‐2023).

## CONFLICT OF INTEREST STATEMENT

The authors declare that they have no conflicts of interest.

## ETHICS STATEMENT

This study was approved by the Ethics Commission of Northwest and Central Switzerland (BASEC ID number: 2022–02297).

## PATIENT CONSENT STATEMENT

Oral/written informed consent was obtained from all individual participants included in the study.

## Supporting information


Data S1

Figure S1

## Data Availability

The data that support the findings of this study are available on request from the corresponding author. The data are not publicly available due to privacy or ethical restrictions.
