# Supplementary material for: Listening to patients: A qualitative study on diagnostic delay, coping strategies and stigma in early‐onset colorectal cancer
Source: Colorectal Dis. 2025 Oct 28;27(11):e70285. doi: 10.1111/codi.70285 (PMC12559872; doi:10.1111/codi.70285)
Supplement: Supplementary file 1 — Data S1 [file CODI-27-0-s002.docx]

# Supporting Information

1. Table S1: **Participant characteristics**
   Figure S1: **A. Diagnostic Delay–** Wrongly diagnosed with benign proctological diseases
   Figure S2: **B. Coping through Support-Seeking and Regaining Control –** Delivering Cancer Diagnosis in Presence of Relatives
   Figure S3: **B. Coping through Support-Seeking and Regaining Control** – Proactive Action: “Fighting to Move Things Forward”
   Figure S4: **B. Coping through Support-Seeking and Regaining Control** – Overwhelmed by the System: “Too Fast to Process”
   Figure S5: **B. Coping through Support-Seeking and Regaining Control** – Overlooked Concern: Fertility and Family Planning
   Figure S6: **C. Living with Cancer: From Stigma to Post-Traumatic Growth –** Alopecia
   Figure S7: **C. Living with Cancer: From Stigma to Post-Traumatic Growth** – Protecting Children

**P07:** *“I had blood in my stool. So, I went to the doctor and they thought I had haemorrhoids.”*

**P19:** *“…because for a few weeks now I have been seeing blood, small amounts, but blood in my stool… Fresh blood and, yes, I wanted to discuss that with [my family doctor]. And he did […] this palpation test from behind and said, yes, he could feel something there, yes, according to age and so on, he would test for haemorrhoids… [later in the interview] there was gallons of blood coming out. I went to the doctor there, yes. OK. Everything okay. Thanks. Yes. Prescribed an ointment.”*

**P12:** “*At some point I went to my family doctor and brought it up. And I was diagnosed relatively quickly with haemorrhoids.”*

**P02:** *“Originally, I had already noticed blood in my stool […] I went to see a gastroenterologist. He then interpreted it as a fissure and months passed while I treated it with the ointment.”*

**P06:** “*I had slight traces of blood in my stool and then I contacted my family doctor again and went back. He then said that it was nothing, that it was normal and actually sent me back again with a medication that I should take for haemorrhoids, so that I could have a better bowel movement.”*

**P20****:** *“I was actually bleeding out the back. And yes, at first they didn't know what it was. They thought it was haemorrhoids.”*

**P12:** *“Hmm, I was very lucky to have my best friend with me […] I didn't realise or understand much at that moment.”*

**P05:** “*I'll say now, the conversations right after I woke up, after the colonoscopy were at least a little difficult because I can't remember, just, yes, selectively.”*

**P03:** *“After the colonoscopy […], I was there with my wife. That was quite good […] I was told by the doctor, they had found a fairly large tumour in my colon.”*

**P19:** *“Yes, it was bad and... My wife was there... Yes. Yes, we got back on track relatively quickly. That's how it is now and now we have to get through it and we'll manage.”*

**P20:** *“That's why I got [my mother] first and then my boyfriend too.”*

**P03:** *“Even if everyone says two weeks won’t change anything, but in your head, it starts every minute and that's when I was probably a bit exhausting, [...] and kept asking when [treatments] would start. […], then next week and then you have to wait another week and then I really counted down the days to the tumour conference and now we have made a plan for you, a therapy plan, then come by and then maybe the day after tomorrow will fit. Oh no, maybe next day, oh no, well. I've always fought myself through so that everything went straight away, but I think if I hadn't done that, it would have taken quite a long time, so then it wouldn't have started until weeks later. I also said I'd take chemotherapy tablets straight away. Yes, then you have to come by again to see the doctor and then I called again because the appointment was in a week or two, no, well, then come by again tonight.”*

**P09:** *“I was lucky. Immediately after this day, I registered at [the hospital]. And I think I called there. I wanted to have an appointment quickly. At first, they wanted to send me a letter and then I said, hey, this is taking too long. Then I got an email, and I had nothing else to do. So, I quickly organised an appointment. On Tuesday I called the doctor. And then I had the letter on Friday. You just have to organise it yourself […] If I hadn't done that, it would have taken another three years.”*

**P12:** *“I had one doctor's appointment after another, and it all bombarded me. I don't think there was enough time either to really sit and tell myself what was happening.”*

**P16:** *“*S*ome of them wanted to do the liver biopsy on the same day as the colonoscopy. Then I said, no.”*

**P12:** *“It took us a long time, to understand what radiation would have meant in that case. Precisely that I couldn't have children […] Once we understood that, I always say we… were very, very scared and started to defend ourselves. […] we always wanted to start a family […] and the decision is actually made for you. […] And I was incredibly lucky with […], the doctor who operated on me. […], who then realised that it was a huge topic and a huge problem for us and who took everything into account to prevent the radiation. He made […] connection to the fertility clinic […] they took my eggs there.”*

**P01:** *“She thinks I still have time […], she'll talk to the doctor again […], and she would definitely recommend it, and the surgeon then said, no […] They said that most patients are older, and that's not an issue at all.”*

**P08:** *“I didn't have chemotherapy, so there was no hair loss.”*

**P01:** *“I haven't lost any hair or anything due to the chemotherapy.”*

**P07:** *“Well, I haven't lost any hair.”*

**P04:** *“I'm not really going to lose all my hair.”*

**P02:** *“So, the hair has perhaps become a little thinner […]. But then there's nothing that you could have seen.”*

**P14:** *“I tolerated the chemotherapy relatively well, didn't lose any hair.”*

**P17:** *“[my chemotherapy treatment] doesn't result in any hair loss or anything else.”*

**P11:** “*I did not want the* *children to find out.*”

**P07:** *“[the daughter] sees that I have the pump, because I go home on Saturday, Sunday and I have the pump, and she realises that something is wrong.*”

**P18:** *“No. It's more like, how and what do I tell the children?”*
