# Supplementary material for: Listening to patients: A qualitative study on diagnostic delay, coping strategies and stigma in early‐onset colorectal cancer
Source: Colorectal Dis. 2025 Oct 28;27(11):e70285. doi: 10.1111/codi.70285 (PMC12559872; doi:10.1111/codi.70285)
Supplement: Supplementary file 2 — Figure S1 [file CODI-27-0-s001.docx]

# Supplementary

1. Interview guide

**Research questions:**

HOW do EOCC patients perceive the diagnosis and its challenges?

WHICH unmet needs and WHAT perspectives do EOCC patients have?

WHAT questions to clinical and basic researchers do EOCC patients have? (Can we do more?)

**Semi-structured interview guide**

**0. Introduction**

- Welcome and introduction
- Explanation of the purpose of the study
- Clarification of questions (declaration of consent)

**1. Background information**

- Asking for demographic data (age, gender, occupation, etc.)
- Inquiring about the time of diagnosis

**2. Symptoms interpretation**

- How did you interpret your symptoms before you were diagnosed?
- How did you deal with your symptoms before you were diagnosed?
- How did you deal with assumptions or fears about the possible cause of your symptoms?

**3. Dealing with diagnostic investigations**

- How did you feel during the diagnosis?
- How did you feel during this time?
- How did you cope during this time?
- Would you have retrospectively used low-threshold preventive services (e.g. app/score for calculating the risk with corresponding recommendation for/against further diagnostics based on your symptoms before diagnosis?
- In your opinion, how important are simpler examination methods beyond the classic colonoscopy?
- Would you have had a blood test to determine the risk?
- Would you have had a simple mucosal biopsy taken from the mouth or rectum?

**4. Thoughts after the diagnosis**

- How did you react when the diagnosis was confirmed?
- What thoughts went through your mind?
- How did you personally interpret the diagnosis?

**5. Reactions of those around you**

- How did your environment (family, friends, acquaintances, professional environment) react to the diagnosis?
- How has the behaviour of those around you changed?
- How have they changed?

**6. Influence of the diagnosis on their everyday life**

- How has the diagnosis affected your daily life?
- How have you adapted? (Perspectives, goals or priorities)
- Do you feel that your symptoms and diagnosis were not taken seriously in the past?
  - If yes, how did you perceive this?
  - If no, why not?

**7. Experienced limitations/restrictions**

- What limitations have you experienced due to radiation?
- What restrictions have you experienced due to chemotherapy?
- How has life with a stoma changed for you?

**8. Body image and fertility**

- How has your body image changed as a result of the illness and treatment?
- If sport has played a major role in your life to date, how has the diagnosis affected your sport?
- What challenges or difficulties did you experience in relation to your fertility and sexuality?

**9. Influence of insurance model and status**

- What influence did your insurance model and status have on your behaviour in dealing with the disease?

**10. Stigmatisation**

- Did you feel stigmatised because of the radiation?
- Did you feel stigmatised because of chemotherapy?
- Did you feel stigmatised because of the ostomy?

**11. Support and hope**

- What moments or experiences have helped you deal with the diagnosis?
- What gave and gives you hope during treatment?

**12. Closure**

- What would you wish for yourself?
- What kind of support would you have liked during the diagnosis?
- What kind of support would you have liked during treatment?
- What would you like to see from the healthcare system, healthcare professionals and science in the future?
- Where do you think researchers should put the most effort into the further development of treatment options in the future?
  - Better early detection?
  - More minimally invasive surgical procedures?
  - Better (more intensive/effective) chemotherapies?
  - Organ-preserving therapy methods?
  - Function-preserving therapy methods?
  - Better psychosocial support during the treatment process?

**13. Conclusion**

- Thanks to the participants
- Offering further information or resources, if desired
- Farewell

1. Research Team and Reflexivity

The core research team (JK/ST/HG/MVS/SE) consisted of surgeons working in the department of surgical oncology at Clarunis Basel. JK is a senior consultant surgeon with a strong academic interest in improving healthcare team communication and collaboration. She brings a unique perspective to the team, as her work often intersects surgical expertise with the complexities of interpersonal dynamics in the operating room and broader clinical environments. Passionate about enhancing team-based care, JK leverages qualitative research methodologies to uncover actionable insights that optimise both clinical outcomes and patient experiences. ST is a senior physician specialising in visceral surgery, with a focus on colorectal surgery. Her research interest lies in advancing the paradigm of personalised medicine in treating colorectal cancer by integrating a patient-centred approach. Of particular interest to her is exploring the expectations and treatment preferences of different patient demographics, notably younger individuals, and understanding how these groups navigate and perceive the healthcare system during their cancer journey. HG recently completed her general surgery residency and is concerned about the rising incidence of cancer among patients in her age group. She is particularly interested in the socio-economic impacts a cancer diagnosis has on this age group, which may differ from those experienced by patients over the age of 50. MVS is deputy head of colorectal surgery of Clarunis and has a strong interest in improving colorectal cancer care especially for affected younger patients. He is the PI of a larger prospective multicentre study which will develop a risk-based screening tool for early onset colorectal cancer (INDICATOR). In his over 20 years working as a surgeon, he has witnessed the dramatic increase of early-onset cases which led to his initiative to improve early detection. SE is an attending surgeon specialising in pancreatic cancer, where she has also observed a concerning rise in younger patients being diagnosed with this disease. Her research on SUMO proteins aims to explore potential molecular factors contributing to this trend, which may offer insights into the underlying causes. SE is also increasingly aware of the need for a more patient-centred approach that considers the unique challenges younger patients face, such as fertility preservation and the socio-economic impact of their diagnosis.

1. Table S1: **Participant characteristics**

| **Demographic and clinical characteristics** | N=19 | |
| --- | --- | --- |
| Gender | abs.* | % |
| Male | 12 | 63 |
| Female | 7 | 37 |
| Age at diagnosis (years) | abs.* | % |
| 30-39 | 7 | 37 |
| 40-44 | 7 | 37 |
| 45-49 | 5 | 26 |
| Localisation | abs.* | % |
| Colon | 8 | 42 |
| Rectum | 11 | 58 |
| Stages | abs.* | % |
| I | 3 | 16 |
| II | 3 | 16 |
| III | 10 | 53 |
| IV | 3 | 16 |

* abs. = absolute value. Stages refer to pre-treatment stages for rectal cancer and non-resected colon cancer and post-resection stages for resected colon cancer.

1. Figure S1: **A. Diagnostic Delay–** Wrongly diagnosed with benign proctological diseases

**P07:** *“I had blood in my stool. So, I went to the doctor and they thought I had haemorrhoids.”*

**P19:** *“…because for a few weeks now I have been seeing blood, small amounts, but blood in my stool… Fresh blood and, yes, I wanted to discuss that with [my family doctor]. And he did […] this palpation test from behind and said, yes, he could feel something there, yes, according to age and so on, he would test for haemorrhoids… [later in the interview] there was gallons of blood coming out. I went to the doctor there, yes. OK. Everything okay. Thanks. Yes. Prescribed an ointment.”*

**P12:** “*At some point I went to my family doctor and brought it up. And I was diagnosed relatively quickly with haemorrhoids.”*

**P02:** *“Originally, I had already noticed blood in my stool […] I went to see a gastroenterologist. He then interpreted it as a fissure and months passed while I treated it with the ointment.”*

**P06:** “*I had slight traces of blood in my stool and then I contacted my family doctor again and went back. He then said that it was nothing, that it was normal and actually sent me back again with a medication that I should take for haemorrhoids, so that I could have a better bowel movement.”*

**P20****:** *“I was actually bleeding out the back. And yes, at first they didn't know what it was. They thought it was haemorrhoids.”*

1. Figure S2: **B. Coping through Support-Seeking and Regaining Control –** Delivering Cancer Diagnosis in Presence of Relatives

**P12:** *“Hmm, I was very lucky to have my best friend with me […] I didn't realise or understand much at that moment.”*

**P05:** “*I'll say now, the conversations right after I woke up, after the colonoscopy were at least a little difficult because I can't remember, just, yes, selectively.”*

**P03:** *“After the colonoscopy […], I was there with my wife. That was quite good […] I was told by the doctor, they had found a fairly large tumour in my colon.”*

**P19:** *“Yes, it was bad and... My wife was there... Yes. Yes, we got back on track relatively quickly. That's how it is now and now we have to get through it and we'll manage.”*

**P20:** *“That's why I got [my mother] first and then my boyfriend too.”*

1. Figure S3: **B. Coping through Support-Seeking and Regaining Control** – Proactive Action: “Fighting to Move Things Forward”

**P03:** *“Even if everyone says two weeks won’t change anything, but in your head, it starts every minute and that's when I was probably a bit exhausting, [...] and kept asking when [treatments] would start. […], then next week and then you have to wait another week and then I really counted down the days to the tumour conference and now we have made a plan for you, a therapy plan, then come by and then maybe the day after tomorrow will fit. Oh no, maybe next day, oh no, well. I've always fought myself through so that everything went straight away, but I think if I hadn't done that, it would have taken quite a long time, so then it wouldn't have started until weeks later. I also said I'd take chemotherapy tablets straight away. Yes, then you have to come by again to see the doctor and then I called again because the appointment was in a week or two, no, well, then come by again tonight.”*

**P09:** *“I was lucky. Immediately after this day, I registered at [the hospital]. And I think I called there. I wanted to have an appointment quickly. At first, they wanted to send me a letter and then I said, hey, this is taking too long. Then I got an email, and I had nothing else to do. So, I quickly organised an appointment. On Tuesday I called the doctor. And then I had the letter on Friday. You just have to organise it yourself […] If I hadn't done that, it would have taken another three years.”*

1. Figure S4: **B. Coping through Support-Seeking and Regaining Control** – Overwhelmed by the System: “Too Fast to Process”

**P12:** *“I had one doctor's appointment after another, and it all bombarded me. I don't think there was enough time either to really sit and tell myself what was happening.”*

**P16:** *“*S*ome of them wanted to do the liver biopsy on the same day as the colonoscopy. Then I said, no.”*

1. Figure S5: **B. Coping through Support-Seeking and Regaining Control** – Overlooked Concern: Fertility and Family Planning

**P12:** *“It took us a long time, to understand what radiation would have meant in that case. Precisely that I couldn't have children […] Once we understood that, I always say we… were very, very scared and started to defend ourselves. […] we always wanted to start a family […] and the decision is actually made for you. […] And I was incredibly lucky with […], the doctor who operated on me. […], who then realised that it was a huge topic and a huge problem for us and who took everything into account to prevent the radiation. He made […] connection to the fertility clinic […] they took my eggs there.”*

**P01:** *“She thinks I still have time […], she'll talk to the doctor again […], and she would definitely recommend it, and the surgeon then said, no […] They said that most patients are older, and that's not an issue at all.”*

1. Figure S6: **C. Living with Cancer: From Stigma to Post-Traumatic Growth –** Alopecia

**P08:** *“I didn't have chemotherapy, so there was no hair loss.”*

**P01:** *“I haven't lost any hair or anything due to the chemotherapy.”*

**P07:** *“Well, I haven't lost any hair.”*

**P04:** *“I'm not really going to lose all my hair.”*

**P02:** *“So, the hair has perhaps become a little thinner […]. But then there's nothing that you could have seen.”*

**P14:** *“I tolerated the chemotherapy relatively well, didn't lose any hair.”*

**P17:** *“[my chemotherapy treatment] doesn't result in any hair loss or anything else.”*

1. Figure S7: **C. Living with Cancer: From Stigma to Post-Traumatic Growth** – Protecting Children

**P11:** “*I did not want the* *children to find out.*”

**P07:** *“[the daughter] sees that I have the pump, because I go home on Saturday, Sunday and I have the pump, and she realises that something is wrong.*”

**P18:** *“No. It's more like, how and what do I tell the children?”*
